# Promethazine-induced neuroleptic malignant syndrome under general anesthesia: a case report

**DOI:** 10.3389/fpsyt.2026.1700644

**Published:** 2026-01-28

**Authors:** Peng Liu, Zhi-Min Liao

**Affiliations:** 1 Department of Anesthesiology, West China Second University Hospital Sichuan University, Chengdu, China; 2 Key Laboratory of Birth Defects and Related Diseases of Women and Children (Sichuan University), Ministry of Education, Chengdu, China

**Keywords:** antipsychotic drugs, general anesthesia, neuroleptic malignant syndrome, NMS, promethazine

## Abstract

**Background:**

Neuroleptic malignant syndrome (NMS) is a rare yet life-threatening disease that is often induced by antipsychotic medications.

**Case presentation:**

In this study, we describe a case of a 13-year-old female who developed NMS following promethazine use during general anesthesia. This is expected to provide clinicians, particularly anesthesiologists, with crucial insights that will improve the perioperative identification and management of this condition. The patient manifested classic clinical features of NMS, including hyperpyrexia, autonomic dysfunction, and generalized rigidity. However, the patient’s serum creatine phosphokinase (CPK) levels were within normal limits, demonstrating the heterogeneous presentation of the disorder. The main interventions included drugs targeting to achieve airway control, physical cooling, hemodynamic stabilization, and symptomatic management, which promoted the patient’s gradual recovery.

**Conclusion:**

This case illustrates that due to individual variations, even standard drug dosages may induce neuroleptic malignant syndrome (NMS). Notably, NMS lacks specific laboratory findings, and its diagnosis must rely on clinical symptoms to avoid delays in early identification and treatment.

## Introduction

Neuroleptic malignant syndrome (NMS) is a rare and life-threatening complication, first described in 1960 in case treated with antipsychotic drugs. It has an incidence rate of 0.02–3.23% and a mortality rate of 5.6% (1). Its hallmark clinical features include altered mental status, muscle rigidity, hyperthermia, and autonomic nervous system dysfunction, in some cases accompanied by abnormal laboratory findings such as elevated serum creatine kinase (CK) levels and leukocytosis (2). Generally, the most common feature is the sudden increase or discontinuation of medications such as antipsychotics, antiemetics, or antidepressants within one week, this syndrome is primarily triggered by high-dose antipsychotic medications, with reported incidence rates ranging from approximately 0.167 to 32.6 cases per 1,000 individuals (3–5). Here, we present a case of NMS triggered by repeated preoperative use of promethazine during surgery, without an increase in serum creatine kinase (CK) levels.

Promethazine is a phenothiazine derivative known to function as an histamine H1 receptor antagonist and dopamine receptor antagonist (6). The drug is applied in the control of allergy or anti-emetic purposes. Its side effects include respiratory depression, coma, extrapyramidal reactions, and neuroleptic malignant syndrome. The patient presented with more severe side effects—neuroleptic malignant syndrome. The following keywords were used: “neuroleptic malignant syndrome,” “malignant syndrome,” “antipsychotic malignant syndrome,” “promethazine,” and “general anesthesia” to identify for relevant literature reports but did not identify any documented cases of NMS associated with promethazine during general anesthesia surgery.

## Case presentation

A 13-year-old female weighing 29kg with a height of 145 cm was diagnosed with small cell sarcoma (Ewing’s sarcoma) through biopsy of a head and neck mass. A bone marrow biopsy revealed a neurogenic tumor (neuroblastoma)? with bone marrow infiltration. The patient had no prior history of other organic diseases or psychiatric disorders; her parents were in good health and one elder sister, and there was no significant family or genetic history. The parents underwent surgeries under general anesthesia without complications. Laboratory tests identified the following blood counts: Alanine aminotransferase (ALT): 34 U/L, Aspartate aminotransferase (AST): 51 U/L, Creatinine (Cr): 28 μmol/L, Potassium (K+): 4.2 mmol/L, Calcium (Ca): 2.51 mmol/L, Serum Iron (SI): 32.5 μmol/L.

The initial diagnosis following admission was a malignant tumor of the neck, suspected to be a neuroblastoma or Ewing’s sarcoma. The patient received chemotherapy to control tumor growth (topotecan + cyclophosphamide), transfusion of hemoglobin and platelets for the treatment of anemia, and etamsylate was administered to alleviate bleeding. During chemotherapy, the child experienced severe vomiting, which was treated with 12.5mg of promethazine intramuscularly. The patient developed thrombocytopenia and anemia during treatment, which was controlled through 8 platelet transfusions before surgery, and prophylactic promethazine 12.5mg given before each transfusion. Due to the need for prolonged chemotherapy, the patient was scheduled to receive elective implantation of a right subclavian infusion port under general anesthesia. Surgery was conducted 3 days following the last dose of promethazine.

**Figure 1:**
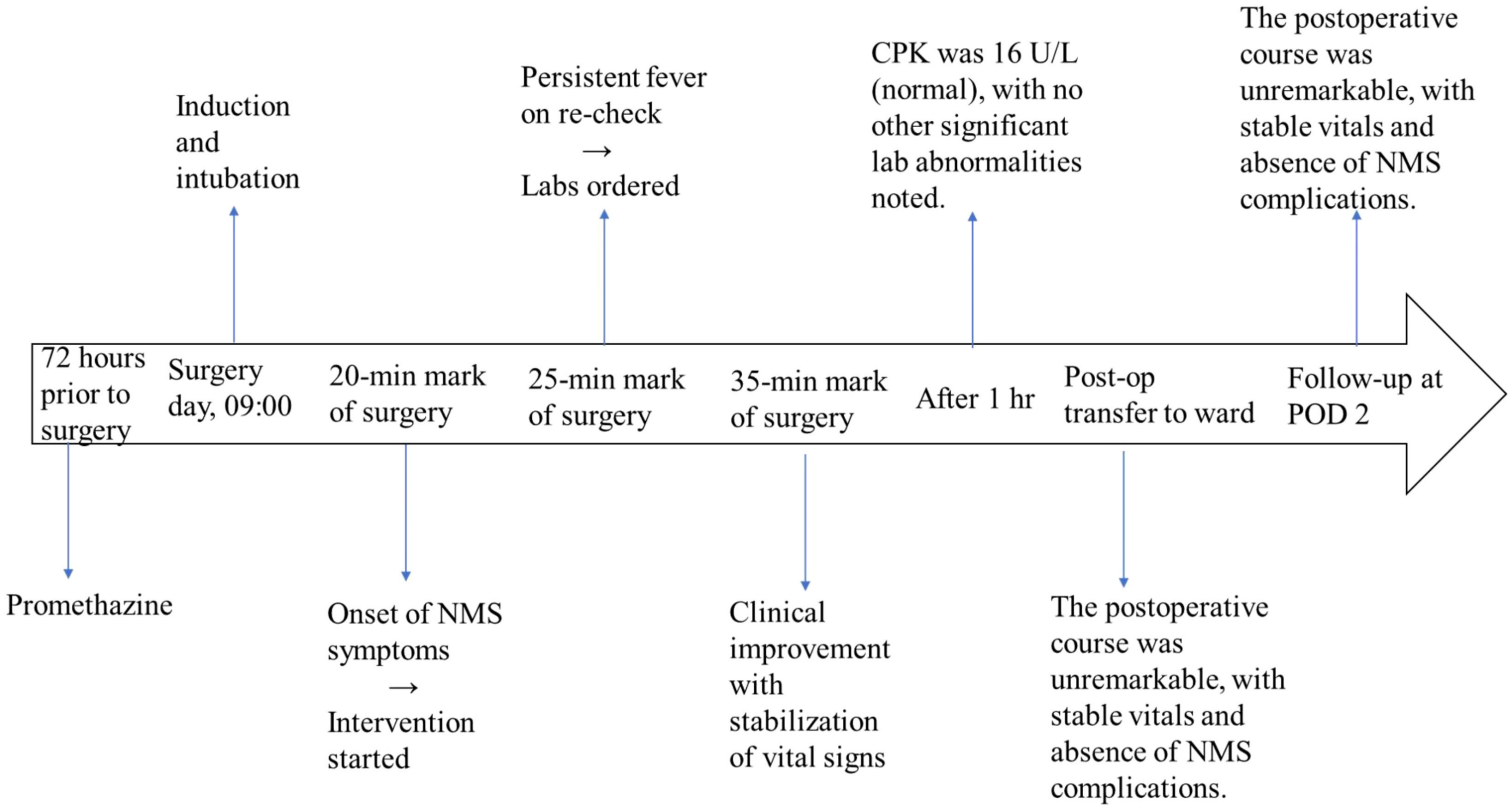
Clinical course.

The patient entered the operating room at 9:00 AM following fasting for 10 hours. Fentanyl 40 mcg and propofol 60 mg were administered, followed by the insertion of a No. 2.5 double-lumen laryngeal mask. Anesthesia was maintained with 3% sevoflurane. During the operation, further intermittent doses of fentanyl (30 mcg and 20 mcg) were administered. Approximately 20 minutes after the operation, the tidal volume was decreased, accompanied with a gradual rise in end-tidal CO_2_ (peaking at 62 mmHg), increase in the heart rate to 140 bpm, progressive elevation of blood pressure, and chest tightness and abdominal wall muscles. Temperature measurements were as follows: 38.3°C at the abdominal wall, 37.3°C at the chest wall, and 36.3°C at the forehead. Base on the above findings, malignant hyperthermia (MH) or neuroleptic malignant syndrome (NMS) was suspected. Sevoflurane was immediately discontinued, and intermittent boluses of propofol (approximately 30 mg) were administered. Ice packs were placed on the neck and axillae to facilitate physical cooling, and 500 mL of lactated Ringer’s solution was infused to achieve volume expansion. The surgical team was informed, and all efforts were made to expedite the procedure. After about 5 minutes, repeat temperature measurements showed 39.2°C at the abdominal wall, 38.8°C at the chest wall, and 38.3°C at the forehead. Blood samples were immediately collected for CBC, biochemistry, creatine phosphokinase (CPK) testing, and arterial blood gas (ABG) analysis. ABG results: PH 7.474, PCO_2_ 30.5mmHg, BE-1.2 mmol/L, HCO_3_^−^ 22.4 mmol, K^+^ 4.3 mmol/L, Na 136 mmol/L, Glu 13.6 mmol/L, PaO2 198.5mmHg, HCT 24%, Hb 83 g/L, SO2 99.8%, LaC 0.72 mmol/L, Ca^2+^ 1.11 mmol/L. Based on these findings, malignant hyperthermia was temporarily ruled out, and the physical cooling measures was continued. The surgical operation was completed within 10 minutes, during which the patient’s vital signs, temperature, and EtCO_2_ gradually stabilized at 43 mmHg. The patient regained consciousness approximately 10 minutes postoperatively. Following a 30-minute observation period after the removal of the post-laryngeal mask, during which the heart rate, blood pressure, and temperature before discharge remained stable, the patient was safely transferred back to the general ward. Laboratory test results after 1 hour showed: Creatine kinase isoenzyme <0.18 μg/L, creatine phosphokinase 16 U/L, K^+^ 4.0 mmol/L, Ca^+^ 2.23 (2.23−2.25 mmol/L), serum iron 33 μmol/L (7.8−32.2), white blood cells 2.9 (4.1−11.0 x 10^9^/L), hemoglobin 75 (110–150 g/L), Glu 3.82 (3.9−11.1 mmol/L). Malignant hyperthermia was ruled out, and the clinical diagnosis was neuroleptic malignant syndrome. There was no complications associated with neuroleptic malignant syndrome during postoperative follow-up. A clinical course is shown in **Figure 1**.

## Discussion and conclusions

Studies have demonstrated that promethazine may precipitate NMS through dopamine receptor antagonism, which disrupts dopaminergic-cholinergic equilibrium—a mechanism grounded in established pathophysiology (7, 8). The present case is notable for documenting NMS onset specifically during general anesthesia.

Th present patient repeatedly used promethazine over a one-month period to prevent platelet transfusion allergies and as an antiemetic, with each dose being 12.5 mg. This treatment is a component of the routine protocol in the pediatric hematology-oncology department of our hospital. The incidence of NMS is extremely low, and its onset is associated with high-dose use of antipsychotic-related medications or sudden discontinuation. However, there are reports that even low doses of antipsychotic-related drugs can induce NMS, suggesting that the risk of developing NMS may be attributed to individual susceptibility rather than being absolutely dose-dependent on DRBA (dopamine receptor blocking agents) (9, 10). Our patient used promethazine irregularly and multiple times in the past month, with the last dose taken three days ago. The patient developed hyperthermia, rigidity of the chest and abdominal wall muscles, tachycardia, and hypertension following intravenous-inhalation combined anesthesia. As an anesthesiologist, my primary consideration was the development of malignant hyperthermia. Following interventions such as the discontinuation of the inhalational anesthetic, high-flow pure oxygen hyperventilation, and physical cooling—along with arterial blood gas analysis and creatine kinase testing—a diagnosis of malignant hyperthermia was ruled out. This treatment alleviated the patient’s symptoms. Based on the DSM-V criteria, our team’s diagnosis is NMS.

The pathophysiology of NMS is considered to be a state of central dopaminergic hypofunction, caused by a sudden and severe reduction of dopamine D2 receptor activity in the central nervous system (11). This disrupts the dopaminergic-cholinergic balance in the striatum, resulting in relative cholinergic hyperactivity and a cascade of homeostatic changes resembling Parkinsonian symptoms, such as dopamine depletion in the tuberoinfundibular pathway causing thermoregulatory dysfunction and fever, alterations to the nigrostriatal pathway inducing muscle rigidity, and even mental status impairment due to autonomic nervous system dysfunction (5). Currently, the incidence of NMS is low, there is no standardized diagnostic or treatment protocol. Since the mid-1980s, several attempts have been made to establish standardized NMS diagnostic criteria, such as those proposed by Levenson and colleagues (12), DSM-V (2), and Addonizio and colleagues (13), but no unified standard has been achieved. The most widely accepted diagnostic criteria for NMS is that proposed by an international expert panel (14).

The patient’s laboratory tests had no significant abnormalities, and CPK was not significantly increased. In previous case reports (15, 16), no CPK elevation was reported, which further confirmed the heterogenous manifestations of this condition. The diagnosis of typical NMS relies on the presence of four core features: hyperthermia, rigidity, autonomic dysfunction, and altered mental status, supported by laboratory findings such as elevated CPK, atypical NMS is defined by the presence of three of these four symptoms. Our case, strictly speaking, falls within the category of atypical NMS (17, 18). In some cases, NMS with elevated CPK was postulated to involve more severe skeletal muscle symptoms, such as rhabdomyolysis (8). Traditionally, patients scheduled for elective surgery are advised to fast after 22:00 the night before to prevent perioperative adverse events like aspiration, potentially inducing a state of dehydration. Several factors such as stress, dehydration, high environmental temperatures, emotional disturbances, and high-dose antipsychotic medications (19, 20), have been identified to increase the risk of NMS (dehydration, stress). Considering the presentation and diagnostic criteria of the present case, the diagnosis of NMS was made. Previous studies have suggested that opioids can influence perioperative NMS development.

For anesthesiologists, the most important aspect of perioperative NMS is its differential diagnosis from MH, since they share symptoms such as hyperthermia, muscle rigidity, and autonomic nervous system dysfunction. The pathogenesis of MH comprises impaired calcium ion homeostasis in skeletal muscle cells. Once MH occurs, a rapid increase in sarcoplasmic calcium ions can trigger hypermetabolic response and rhabdomyolysis (21, 22). However, in this case, the patient’s calcium levels were consistently within normal levels. Second, the core diagnostic criteria for malignant hyperthermia include hyperkalemia and elevated creatine kinase (23), yet this patient’s laboratory results showed no significant abnormalities. Third, MH is an inherited hypermetabolic syndrome, mostly transmitted in an autosomal dominant pattern. Both parents of the child had previously undergone general anesthesia without any history of MH-related conditions (24). Consequently, in this case, the possibility of MH was ruled out following symptomatic treatment and symptom resolution.

The differentiation of serotonin syndrome (SS) is a critical consideration. SS is triggered by an excess of serotonin (5-HT) in the central nervous system, typically resulting from the use of serotonergic agents or other substances that increase serotonin levels. Subtle distinctions between SS and NMS exist in terms of mental status, muscular abnormalities, and medication history. In SS, mental status changes include agitation, confusion, and restlessness, whereas patients with NMS frequently present with stupor or mutism. Regarding muscular manifestations, NMS is characterized by “lead-pipe” rigidity, while SS typically features myoclonus and cramps. Additionally, SS commonly involves gastrointestinal symptoms such as diarrhea and vomiting, which are not typically associated with NMS (25). For treatment, benzodiazepines and serotonin antagonists constitute the standard therapeutic approach for SS (26). These distinct clinical presentations and medication histories are crucial for differentiating between the two conditions.

**Figure 2:**
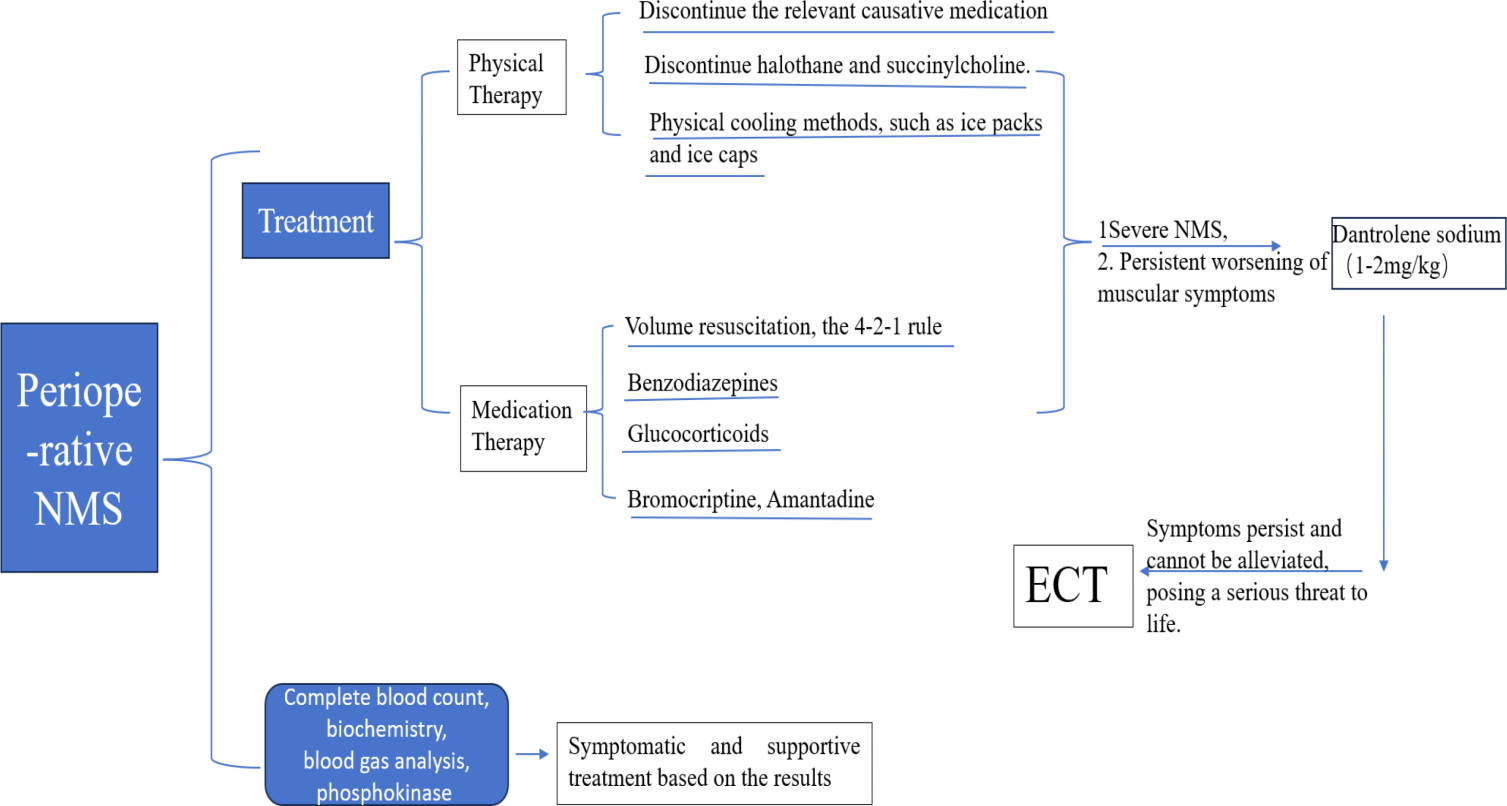
A treatment flowchart.

A diagnosis of NMS in the perioperative period necessitates an immediate intervention. Currently, there is no definitive, authoritative expert consensus on the treatment plan, and treatment is based on the combination of experience and theoretical consideration. The first step is to discontinue the use of relevant dopamine receptor-blocking agents. Second, interventions that aim at stabilizing hemodynamics and correcting electrolyte imbalances are adopted. Third, cooling measures are immediately initiated, such as placing ice packs in areas with major blood vessels like the armpits, groin, and neck, applying an ice cap to the head, adjusting the operating room temperature, and using alcohol wipes for physical cooling. Currently, evidence supporting the use of nonsteroidal anti-inflammatory drugs (NSAIDs) as a cooling intervention is insufficient. Fourth, aggressive volume resuscitation is applied to prevent acute renal failure, especially considering that most NMS patients are dehydrated during the acute phase of the disease (9, 24). In addition, urine output is monitored, maintaining it at 0.5–1 mL/kg/h. Recent reports have observed that alkalinized fluids or even a bicarbonate load can potentially prevent renal failure (27), NMS-induced muscle rigidity can precipitate rhabdomyolysis, causing calcium dysregulation characterized by hypocalcemia in the oliguric phase and hypercalcemia in the diuretic phase (25, 26). Fifth, specific medications are recommended to treat patients with NMS. Benzodiazepines (e.g., midazolam 0.05–0.075 mg/kg or lorazepam 1–2 mg) may be administered to indirectly enhance dopaminergic activity and accelerate patient recovery (24). Corticosteroids, which possess dopaminergic and lysosomal membrane-stabilizing properties, may also be used. Dexmedetomidine, which exerts sedative-hypnotic and anxiolytic effects, can also promote NMS treatment. Neurologists and intensive care specialists prefer dopamine receptor agonists (e.g., bromocriptine) and antimuscarinic agents (e.g., amantadine) as pharmacological agents for NMS management (24, 28). Sixth, for severe NMS or persistent/worsening skeletal muscle rigidity, dantrolene sodium (1–2 mg/kg) may be considered (8). Seventh, if the above measures fail to alleviate symptoms, electroconvulsive therapy (ECT)—which delivers small electrical currents to the brain to induce generalized seizures and alter brain chemistry—may be considered (7), although it is not well understood whether it will achieve good clinical benefits. A treatment flowchart is shown in **Figure 2**.

This case report offers three key contributions. First, it describes the successful management of a patient with neuroleptic malignant syndrome (NMS) and the arrest of its clinical progression. Second, the occurrence of NMS during general anesthesia allowed for a structured differential diagnosis of perioperative syndromes presenting with hyperthermia, muscle rigidity, and autonomic dysfunction—providing a practical reference for clinicians. Third, the report raises the hypothesis that general anesthesia-induced central nervous system suppression may have mitigated the severity of NMS, a possibility that warrants further discussion within the medical community. Several limitations should be noted. As a case report, the findings cannot be validated through randomized controlled trials. Some of the interpretations proposed here also lack support from high-grade evidence. Furthermore, the patient met criteria for atypical NMS, and because she was under general anesthesia, assessment of altered mental status—a core diagnostic feature—was not feasible.

In summary, enhanced awareness and early diagnosis of this drug-induced reaction may help to prevent progression to fulminant and minimize fatal NMS episodes. Based on the patient’s positive clinical outcome in this case, we hypothesize that the central nervous system suppression induced by anesthesia may have mitigated the progression of NMS. It must be emphasized that this interpretation remains speculative due to limited supporting evidence at present. Our research team will continue to explore this mechanism in future studies. We hope this case report will serve as a reference to clinicians, especially anesthesiologists, in recognizing the complex and diverse manifestations of neuroleptic malignant syndrome and gain relevant treatment experience.

## Data Availability

The original contributions presented in the study are included in the article/supplementary material. Further inquiries can be directed to the corresponding author/s.
